# Supplementary material for: Expanding the MAPPs Assay to Accommodate MHC-II Pan Receptors for Improved Predictability of Potential T Cell Epitopes
Source: Biology (Basel). 2023 Sep 21;12(9):1265. doi: 10.3390/biology12091265 (PMC10525474; doi:10.3390/biology12091265)
Supplement: Supplementary file 1 [file biology-12-01265-s001.zip › supplementary figures.pdf]

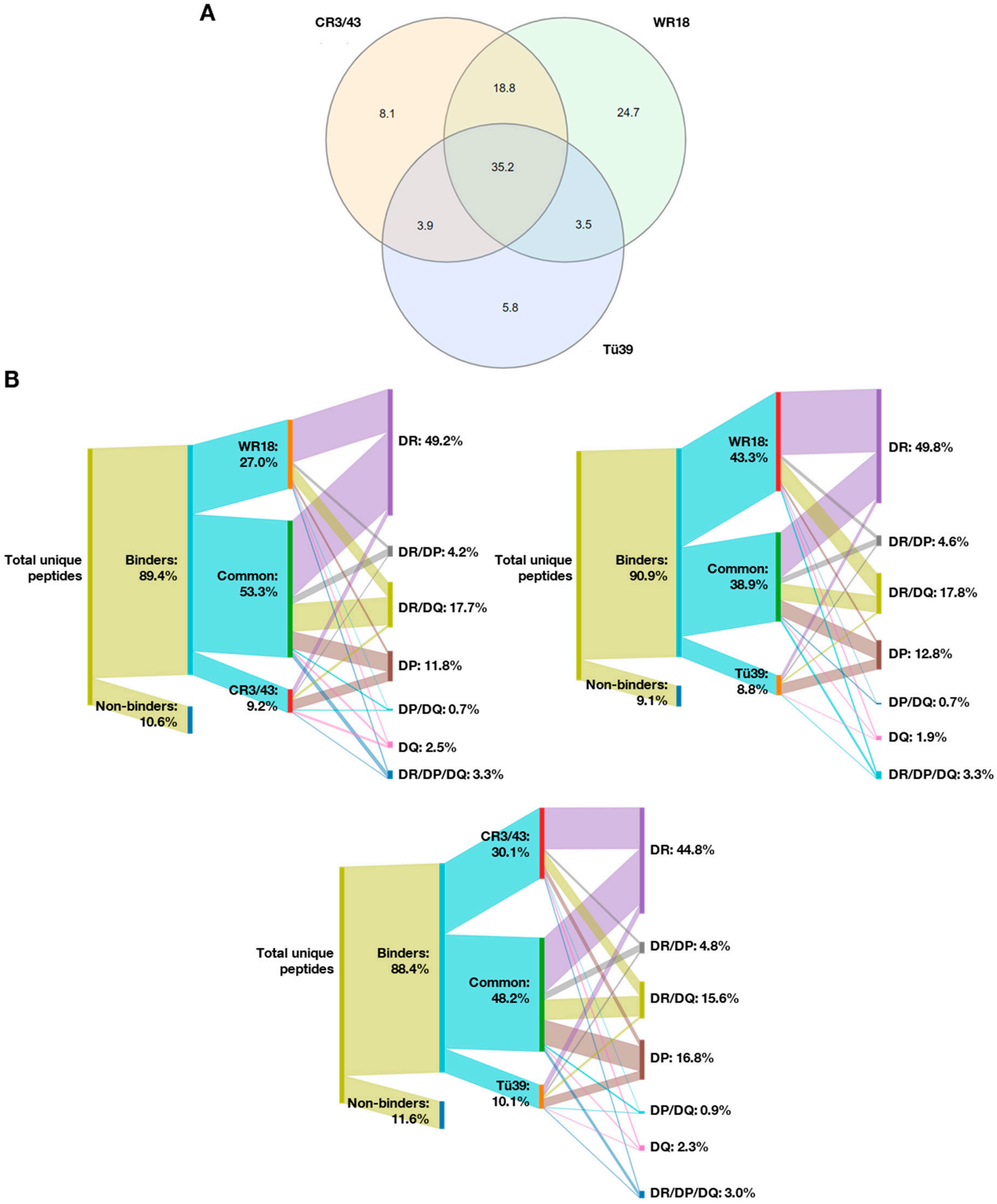

Figure S1. Comparisons of major histocompatibility complex (MHC) II peptide repertoires between MHC-II pan-precipitating antibody clones CR3/43, WR18, and Tü39. (A) Venn diagram depicting percent overlap of total unique peptides identified by MHC-II pan receptor-specific clones CR3/43, WR18, and Tü39. (B) Sankey diagrams of receptor-specific peptide distribution comparisons between clones WR18 versus CR3/43, WR18 versus Tü39, and CR3/43 versus Tü39. The height of the branches is proportional to the distribution. Shown are the percentage of binders and non-binders with respect to the total unique peptide count, and the percentage of peptides identified by each clone alone. "Common" relates to the overlapping unique peptides (i.e. peptides identified by both clones). The distribution of specific MHC-II receptor binders is representative of the total number of MHC-II receptor-specific peptides, and the percent distribution corresponds to the total unique peptide count. Donor 1 is depicted, and representative of all 3 donors tested.

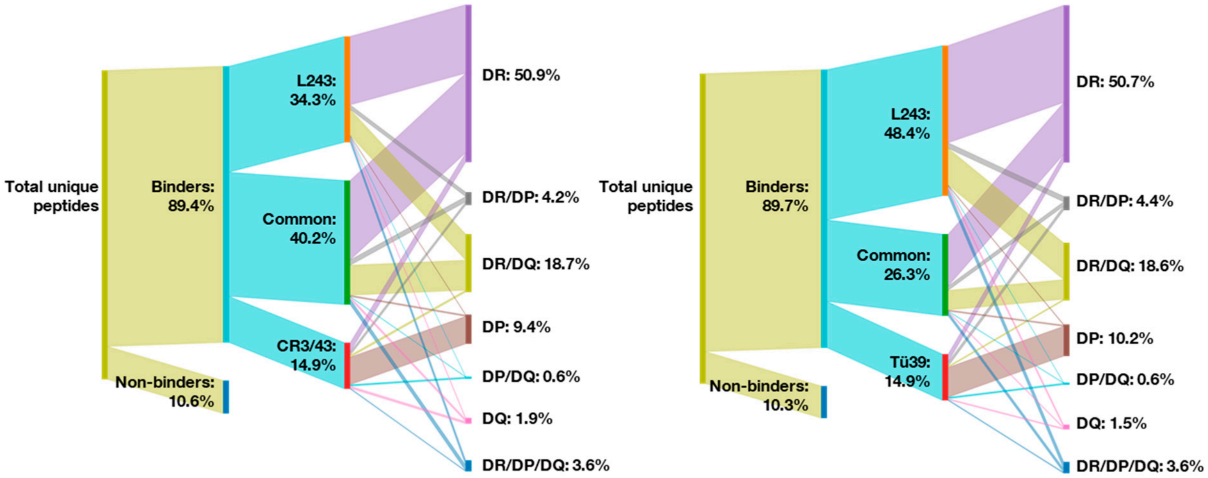

Figure S2. Comparisons of major histocompatibility complex (MHC) II peptide repertoires between human leukocyte antigen (HLA)-DR receptor-specific clone L243 versus MHC-II pan-precipitating antibody clones CR3/43 and Tü39. Sankey diagrams of receptor-specific peptide distribution comparisons between clones L243 versus CR3/43 and Tü39. The height of the branches is proportional to the distribution. Shown are the percentage of binders and non-binders with respect to the total unique peptide count, and the percentage of peptides identified by each clone alone. "Common" relates to the overlapping unique peptides (i.e. peptides identified by both clones). The distribution of specific MHC-II receptor binders is representative of the total number of MHC-II receptor-specific peptides, and the percent distribution corresponds to the total unique peptide count. Donor 1 is depicted, and representative of all 3 donors tested

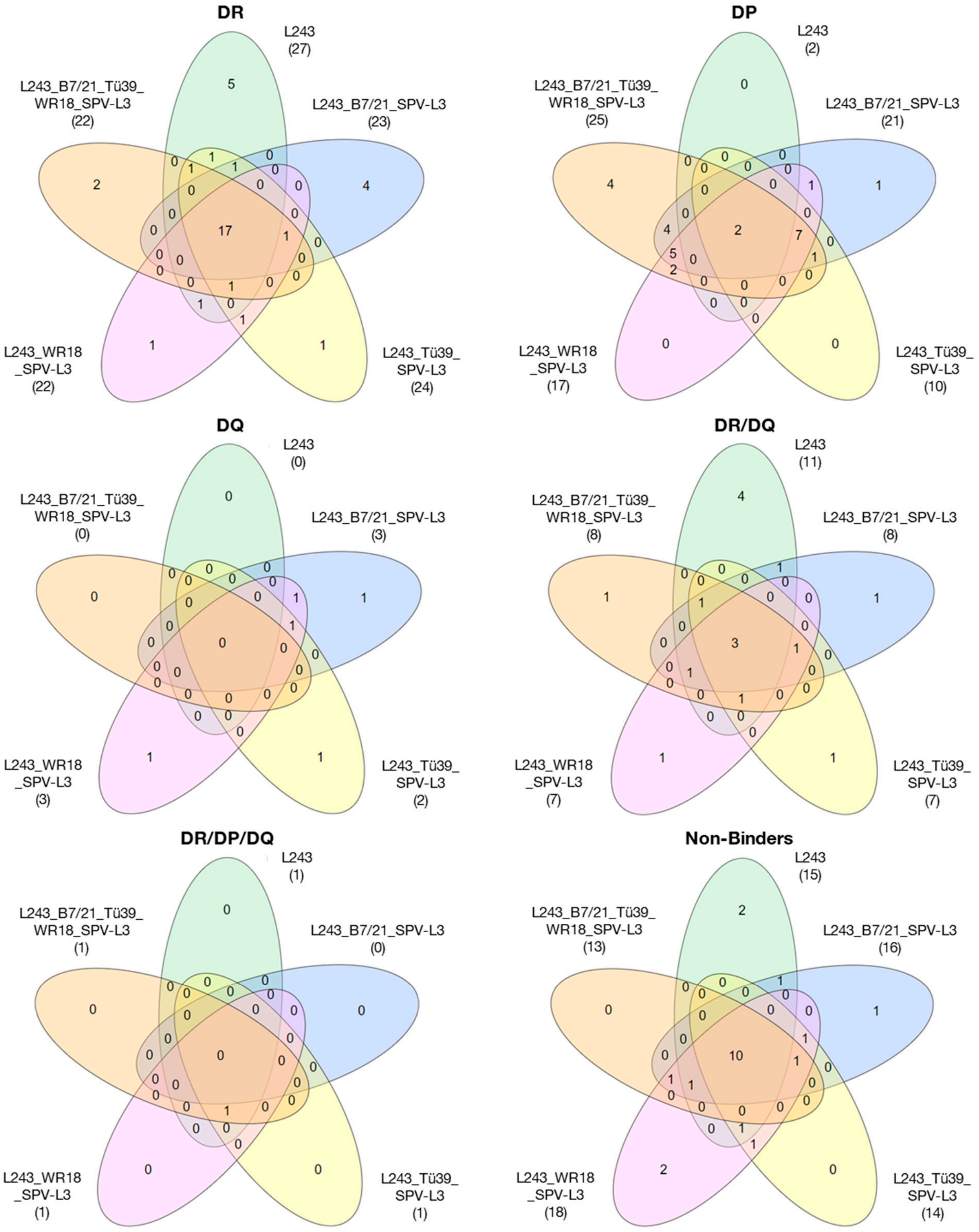

Figure S3. Overlap of identified compound-derived major histocompatibility complex (MHC) II peptides identified using anti-human leukocyte antigen (HLA)-DR clone L243 alone versus various mixed immunoprecipitation (IP) strategies. Identified compound-specific peptide repertoires using clone L243 and each of the mixed IP strategies in the MHC-II-associated peptide proteomics (MAPPs) assay are compared according to NetMHCIIpan-predicted MHC-II receptor binding specificities (annotated as DR, DP, DQ, DR/DQ, DR/DP, and non-binders). Shown is donor 1. The total number of unique peptides are indicated in parenthesis for each sample.

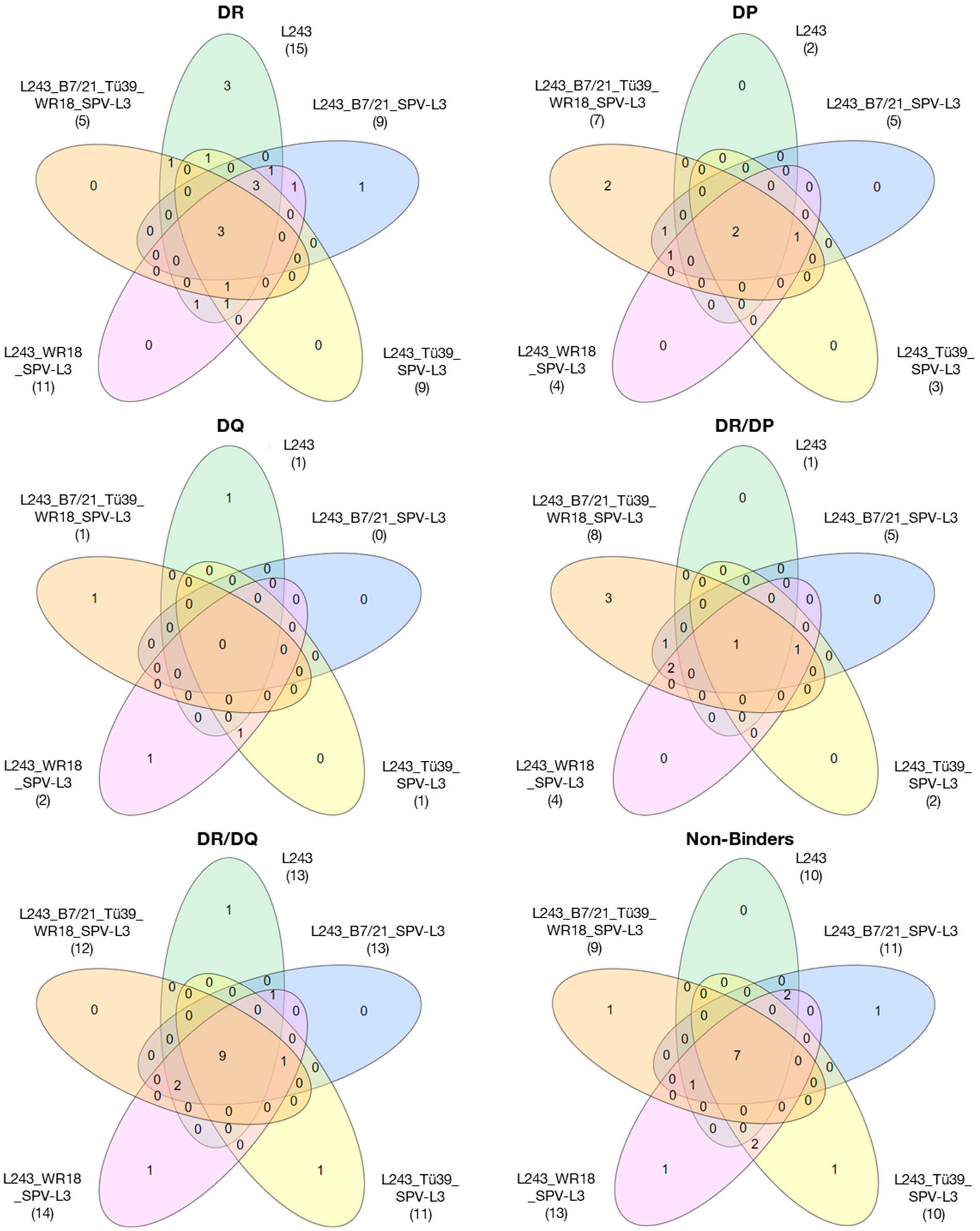

Figure S4. Overlap of identified compound-derived major histocompatibility complex (MHC) II peptides identified using anti-human leukocyte antigen (HLA)-DR clone L243 alone versus various mixed immunoprecipitation (IP) strategies for donor 2. Identified compound-specific peptide repertoires using clone L243 and each of the mixed IP strategies in the MHC-II-associated peptide proteomics (MAPPs) assay are compared according to NetMHCIIpan-predicted MHC-II receptor binding specificities (annotated as DR, DP, DQ, DR/DQ, DR/DP, and non-binders). The total number of unique peptides are indicated in parenthesis for each sample.

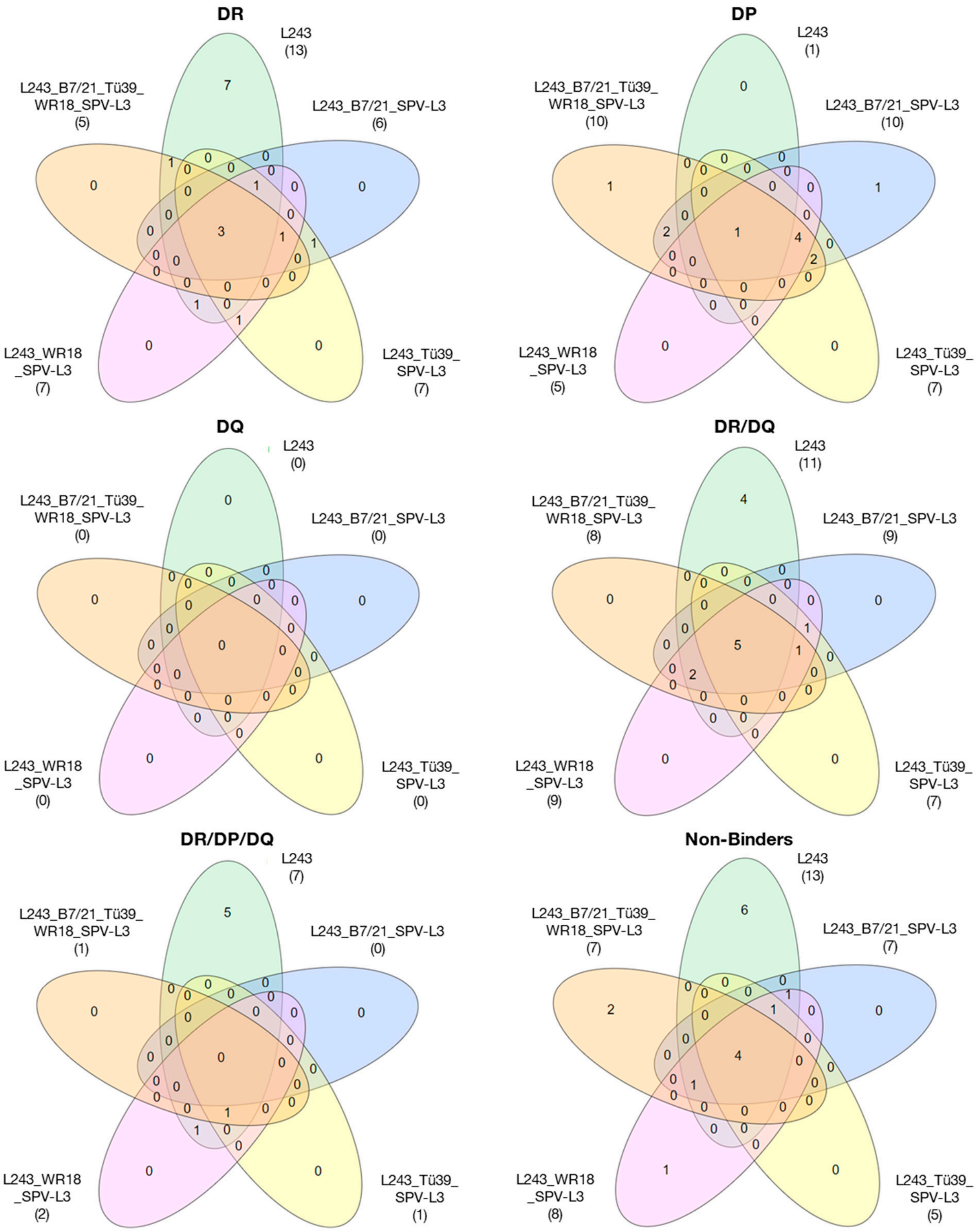

Figure S5. Overlap of identified compound-derived major histocompatibility complex (MHC) II peptides identified using anti-human leukocyte antigen (HLA)-DR clone L243 alone versus various mixed immunoprecipitation (IP) strategies for donor 3. Identified compound-specific peptide repertoires using clone L243 and each of the mixed IP strategies in the MHC-II-associated peptide proteomics (MAPPs) assay are compared according to NetMHCIIpan-predicted MHC-II receptor binding specificities (annotated as DR, DP, DQ, DR/DQ, DR/DP, and non-binders). The total number of unique peptides are indicated in parenthesis for each sample.
